# Transplantation of Directly Reprogrammed Human Neural Precursor Cells Following Stroke Promotes Synaptogenesis and Functional Recovery

**DOI:** 10.1007/s12975-019-0691-x

**Published:** 2019-02-12

**Authors:** Ilan Vonderwalde, Ashkan Azimi, Gabrielle Rolvink, Jan-Eric Ahlfors, Molly S. Shoichet, Cindi M. Morshead

**Affiliations:** 1 Institute of Biomaterials and Biomedical Engineering, University of Toronto, Toronto, Ontario M5S 3E1 Canada; 2 Institute of Medical Science, University of Toronto, Toronto, Ontario M5S 3E1 Canada; 3 Department of Surgery, Division of Anatomy, Donnelly Centre, University of Toronto, Toronto, Ontario M5S 3E1 Canada; 4 New World Laboratories, Laval, Quebec H7V 5B7 Canada

**Keywords:** Transplantation, Stem cells, Cell reprogramming, Synaptogenesis, Functional recovery

## Abstract

Stroke is one of the leading causes of long-term disability. Cell transplantation is a promising strategy to treat stroke. We explored the efficacy of directly reprogrammed human neural precursor cell (drNPC) transplants to promote functional recovery in a model of focal ischemic stroke in the mouse sensorimotor cortex. We show that drNPCs express neural precursor cell markers and are neurally committed at the time of transplantation. Mice that received drNPC transplants recovered motor function, irrespective of transplant vehicle or recipient sex, and with no correlation to lesion volume or glial scarring. The majority of drNPCs found in vivo*,* at the time of functional recovery, remained undifferentiated. Notably, no correlation between functional recovery and long-term xenograft survival was observed, indicating that drNPCs provide therapeutic benefits beyond their survival. Furthermore, increased synaptophysin expression in transplanted brains suggests that drNPCs promote neuroplasticity through enhanced synaptogenesis. Our findings provide insight into the mechanistic underpinnings of drNPC-mediated recovery for stroke and support the notion that drNPCs may have clinical applications for stroke therapy.

**Electronic supplementary material:**

The online version of this article (10.1007/s12975-019-0691-x) contains supplementary material, which is available to authorized users.

## Introduction

Ischemic strokes are the most common type of stroke. Occlusion of cerebrovascular blood flow resulting in a lack of glucose and oxygen delivery to the brain results in rapid cell death and impaired neural function within the affected regions. The resulting functional deficits have a significant impact on an individual’s quality of life and current treatment strategies offer limited success [1–5]. Most available therapies for stroke focus on restoring blood flow and neuroprotection, which have a limited therapeutic window. Cell-based interventions to repair the stroke-injured brain and promote functional recovery have demonstrated some therapeutic efficacy [6–9]. However, a number of challenges including the identification of an appropriate cell type for transplantation that circumvents immune rejection, tumorigenicity, ethical concerns, misguided or misdirected growth, and limited availability in terms of cell isolation and expansion remain [10–13].

Neural precursor cells (NPCs), comprised of neural stem cells and their progeny, have the capacity to differentiate into neural specific cell types, making them good candidates to repair the stroke-injured brain. Although their underlying mechanism of action is not entirely clear, NPCs have demonstrated efficacy in treating several models of stroke, resulting in improved outcomes, including better functional performance, decreased glial scarring, and reduced extent of injury [14–21]. However, harvesting human NPCs is challenging due to their limited availability and location within the brain. Other potential sources of human NPCs are those derived from reprogrammed cells, such as induced pluripotent stem cells (iPSCs), which offer an autologous cell source for transplantation. Unfortunately, iPSCs pose concerns for clinical application because of their acquired pluripotent state during reprogramming in addition to the length of time and complexity required to generate sufficient numbers of cells.

With clinical translation in mind, we examined the therapeutic potential of a population of human cells that have been directly reprogrammed from somatic cells to NPCs*,* without passing through a pluripotent state during reprogramming. We address important considerations that may influence transplant success, including the transplant vehicle [22], and the sex of the stroke-injured mice, which has not been adequately studied to date despite the observation that males and females are differentially responsive to stroke injury [12, 23–26]. Furthermore, we explore the importance of cell survival for recovery and investigate changes in synaptogenesis as a mechanism underlying cell-mediated effects.

Using a preclinical model of cortical stroke, we demonstrate that human directly reprogrammed neural precursor cell (drNPC) transplants delivered during the subacute phase of stroke are sufficient to elicit motor recovery irrespective of recipient sex and transplant vehicle. The observed functional recovery was not correlated with the extent of glial scarring or lesion volumes and did not require long-term xenograft survival. Furthermore, drNPC transplants appear to promote synaptogenesis, as indicated by increased expression of the presynaptic vesicle protein synaptophysin in the ipsilesional hemisphere of transplanted brains. These findings suggest that NPCs may indirectly promote functional recovery by influencing the surrounding tissue, making drNPCs a promising population of cells to treat the stroke-injured brain.

## Materials and Methods

### Study Design

The experimental design was a controlled laboratory experiment. Male and female animals were used and separated into groups via random assignment by a blinded third party until appropriate numbers of samples were achieved for each group. Behavioral analysis was conducted by an observer blinded to the treatment groups. Tissue and cellular outcomes were evaluated by three separate observers blinded to the experimental groups.

For functional analysis, 10–15 mice per group were analyzed. Using a sensitivity analysis on G*Power (version 3.1) with power = 0.8, we determined the treatment effect size to be ~ *f*(*V*) = 0.65 (*η*^2^_partial_ = 0.3) when analyzing all four treatment groups, *f*(*V*) = 0.42 when comparing between drNPC and vehicle groups, and *f*(*V*) = 0.93 when comparing between brains that had surviving drNPCs and those that did not. For tissue outcome comparisons between mice that received drNPCs or vehicle alone, the effect size was always above *d* = 0.9 (Gliosis, 0.97; Lesion Volume, 1.51; Synaptophysin, 1.60) with a power = 0.8. All effect size (sensitivity) calculations were based on Cohen’s *d* [27]. Excluded animals were not considered for power and sensitivity analyses.

### Animals

Immunocompromised Fox Chase SCID/Beige (8–16 weeks old; CB17.Cg-Prkdc^scid^Lyst^bg-J^/Crl; Charles River Laboratories, Wilmington, MA) mice were singly housed on a 12-h light/dark cycle with food and water provided ad libitum for the duration of testing, starting 3 days prior to stroke, until sacrifice. A total of 87 mice (establishment of stroke, *n* = 13, [sex not tracked]; long-term deficit analysis for ET-1 stroke, *n* = 13 [7 males, 6 females]; confirmation of stable measures in long-term testing of naïve mice, *n* = 8 [6 males, 2 females]; therapeutic evaluation of drNPC transplants, *n* = 53 [30 males, 23 females]) were used in this study. Outliers and mice that did not meet our inclusion criteria were removed from the study as described in the supplementary materials.

### Stroke Injury

ET-1 stroke was performed in SCID/Beige mice as previously described [17, 28, 29]. Briefly, the skull was exposed, a small burr hole was drilled at the site of the right sensorimotor cortex at + 0.6 mm anterior and − 2.25 mm lateral to bregma. Mice received a 1-μL injection of ET-1 (Calbiochem, 800 picomolars) 1 mm deep from the surface of the brain at a rate of 0.1 μL/min using a 2.5 μL Hamilton Syringe with a 26 gage, 0.375″ long needle (Hamilton, Reno, NV). The needle was kept in place for 10 min following the injection and then slowly withdrawn.

### drNPC Reprogramming and Preparation

Human bone marrow cells were reprogrammed with transient expression of transcription factors musashi-1 (Msi1), neurogenin-2 (Ngn2), and methyl-CpG binding domain protein 2 (MBD2), as described in detail in the supplementary material. The cells were cultured until they reached ~ 80% confluence in each passage and collected for transplantation after 4–9 passages*.* Sister cultures were prepared for in vitro analysis to characterize the cells using immunocytochemistry and PCR.

### Cell Transplantation

For transplantation, drNPCs were suspended in artificial cerebrospinal fluid (aCSF) or Hyaluronan Methylcellulose (HAMC; supplementary methods) hydrogel (100,000 cells/μL). Cells were transplanted into the stroke site 4 days following stroke with the same surgical procedures used for ET-1-induced ischemia in the sensorimotor cortex. Control animals received 1 μL injections of aCSF or HAMC only.

### Live/Dead Assay

A live/dead assay was performed to determine the percent of surviving cells post-injection through the syringe [30]. Using the same protocol as was used for transplantation, 100,000 cells in 1 μL of vehicle were injected into a well containing 14 μL of warm (37 °C) aCSF at a rate of 0.1 μL/min. Following injection, 15 μL of the live/dead stain solution (0.2% Ethidium Homodimer and 0.05% calcein AM) (L3224, ThermoFisher) was added, followed by a 5-min incubation period after which 220 μL aCSF was added for a final volume of 250 μL. The solution was imaged using an AxioVision Zeiss UV microscope (5× magnification) and images were visualized in FIJI [31]. The percent of live/dead cells was calculated using the “analyze particles” feature on the FIJI software.

### Immunostaining

Fixed tissue and cells were rinsed with 1× phosphate buffered saline (PBS), permeabilized with 0.3% Triton-X100 in 0.01 M PBS for 20 min and blocked in 10% Normal Goat Serum (NGS) with 0.3 M glycine for 1 h at room temperature. Samples were then treated with primary antibodies (Table 1) in 0.01 M PBS and left overnight at 4 °C in a humid chamber. Samples were washed with 1× PBS and exposed to secondary antibodies for 1 h at room temperature (Table 2). The samples were then washed, cover-slipped with mowiol® 4-88 (Sigma-Aldrich), and imaged using an AxioVision Zeiss UV microscope, an Olympus FV1000 confocal point-scanning microscope, or a ZEN Zeiss spinning disk confocal microscope.

**Table 1:** Primary antibodies used in this study

Antibody	Concentration	Product code	Company	Species	Type
Oct4	1:500	sc-5279	Santa Cruz	Mouse	IgG_2b_
Sox2	1:200	ab97959	abcam	Rabbit	Polyclonal
Human nestin	1:500	ABD69	Milipore	Rabbit	Polyclonal
HuNu	1:200	MAB1281	Milipore	Mouse	IgG
STEM121	1:1000	Y40410	Takara	Mouse	IgG1
Ki67	1:200	ab16667	Abcam	Rabbit	Monoclonal
Ki67	1:500	ab15580	Abcam	Rabbit	Polyclonal
TUJ1	1:1000	802,001	Biolegend	Rabbit	Polyclonal
TUJ1	1:1000	T8660	Sigma	Mouse	IgG_2b_
GFAP	1:600	Z0334	Dako	Rabbit	Polyclonal
Olig2	1:200	AB9610	Milipore	Rabbit	Polyclonal
NeuN	1:500	ABN78	Milipore	Rabbit	Polyclonal
Synaptophysin	1:400	AB32127	Abcam	Rabbit	Polyclonal
DAPI	1:10000	D1306	Invitrogen	N/A	N/A
Hoechst 33342	1:1000	H3750	ThermoFisher	N/A	N/A

**Table 2:** Secondary antibodies used in this study

Antibody	Concentration	Wavelength
Goat anti-mouse	1:400	488
Goat anti-rabbit	1:400	488
Goat anti-rabbit	1:400	568
Goat anti-mouse	1:400	555
Goat anti-mouse	1:400	568

### Lesion Volume Analysis

Following cresyl violet staining (see Supplementary methods), serial 20- μm thick coronal sections (200 μm apart) spanning a total of 3–4 mm surrounding the injury site were imaged at 5× magnification using an AxioCam ICc1 camera. The cortical lesion was measured on FIJI using the lasso and polygon tools to outline and quantify the total cortical lesion infarct area, as defined by the area with atypical tissue morphology including pale areas with lost Nissl staining and areas filled with dark pyknotic stained debris [32]. The total volume of the injury was estimated by averaging the area measured in each coronal section and multiplying by the total length of the scar, which was calculated from the number of sections in which the lesion was present.

### Gliosis Measurement and Analysis

A set of serial coronal sections (20 μm thick) immunostained for GFAP^+^ expression were visualized at 5× magnification at 200 μm intervals using FIJI [31]. The total area of cortical GFAP^+^ expression was measured in each section. Measurements were taken from anterior to posterior through the scar and the maximal GFAP expression, as well as the total gliosis volume, was calculated per brain.

### Synaptophysin Imaging and Quantification

All of the images were taken with identical parameters using confocal microscopy on a ZEN Zeiss spinning disk confocal microscope to generate z-stacks comprised of eight optical sections at 0.49 μm per section. The channel exposure was fixed at 1000 ms throughout the imaging of the entire set. Quantification of total synaptophysin-positive pixels per analyzed brain section was conducted by using FIJI [31] to measure the number of positive pixels in the perilesional areas. The mean pixel intensity in two perilesional regions of interest (ROIs) was used to measure the amount of staining (Fig. 7a), as this measure represents the sum of all detected bright pixels (gray values) divided by the total number of pixels within the channel. Imaging, ROI selection, and analysis were conducted by a blinded observer.

### Cellular Characterization and Quantification

Three cell culture wells per biological replicate were stained for each specific antibody and were counted within the field of view in five areas within each well at 20× magnification. The percentage of each cell type was calculated as a percent of all DAPI or Hoechst labeled cells.

For in vivo analysis, coronal sections 20 μm thick at 200 μm intervals were immunostained for HuNu or STEM121 and antibodies found in Table 1. Total numbers of surviving transplanted cells were calculated by extrapolating the average number of surviving drNPCs per section over the total number of sections that contained drNPCs (ranging from 15 to 20 sections). To analyze proliferation, the numbers of Ki67^+^/HuNu^+^ cells were counted in the same representative sections and calculated as a percent of all HuNu^+^ cells. Cell differentiation in vivo post-transplantation was analyzed by immunohistochemistry in brains that had surviving drNPCs.

### Reverse Transcription Polymerase Chain Reaction

Cultured drNPCs were collected into Buffer RL (Norgen Biotek) with β-mercapthenol and then processed according to the manufacturer’s directions using Total RNA Purification Kit (Norgen Biotek — Cat#17200). Cycling conditions consisted of polymerase activation and DNA denaturation (3 min at 98 °C), followed by 35 cycles of 10 s at 95 °C and 30 s at 60 °C. Primer sequences used are listed in Table 3.

**Table 3:** Polymerase chain reaction primer sequences used in this study

Target	Sequence	Expected product size
Sox2	Fwd GGAGCTTTGCAGGAAGTTTG Rev. GGAAAGTTGGGATCGAACAA	460
Oct4	Fwd CTGAGGGTGAAGCAGGAGTC Rev. CTTGGCAAATTGCTCGAGTT	170
Nanog	Fwd AAGGCCTCAGCACCTACCTA Rev. GAGACGGCAGCCAAGGTTAT	979
Nestin	Fwd GCGTTGGAACAGAGGTTGGA Rev. TGGGAGCAAAGATCCAAGAC	327
Pax6	Fwd CAATCAAAACGTGTCCAACG Rev. TGGTATTCTCTCCCCCTCCT	431
Ascl1	Fwd GTCGAGTACATCCGCGCGCTG Rev. AGAACCAGTTGGTGAAGTCGA	220
CD133	Fwd CAGTCTGACCAGCGTGAAAA Rev. GGCCATCCAAATCTGTCCTA	200
Map2	Fwd TCAGAGGCAATGACCTTACC Rev. GTGGTAGGCTCTTGGTCTTT	320
Actb	Fwd TCACCCACACTGTGCCCATCTACGA Rev. CAGCGGAACCGCTCATTGCCAATGG	295
GAPDH	Fwd CTCTGCTCCTCCTGTTCGAC Rev. GCGCCCAATACGACCAAATC	121

### Quantitative Reverse Transcription Polymerase Chain Reaction (RT-qPCR)

Samples were collected into Buffer RL (Norgen Biotek) and processed according to the manufacturer’s directions using Total RNA Purification Kit (Norgen Biotek — Cat#17200). cDNA synthesis was carried out with iScript gDNA Clear cDNA Synthesis Kit (Bio-Rad — Cat# 1725034). RT-qPCR reactions were prepared according to the manufacturer’s directions using SsoAdvanced Universal SYBR Green Supermix (Bio-Rad — Cat# 172-5270). RT-qPCR was carried out on Bio-Rad CFX384 Touch Real-Time PCR System (Bio-Rad). Cycling conditions consisted of polymerase activation and DNA denaturation (3 min at 98 °C), followed by 40 cycles of 10 s at 95 °C and 30 s at 60 °C. All reactions were concluded by incubation at 65 °C and increasing the temperature (at 0.5 °C increments) to 95 °C for melting-curve analysis. Prior to performing relative expression analyses, standard curves were generated for targets (see below) via the serial dilutions of pooled cDNA. In accordance with MIQE (Minimum Information for Publication of Quantitative Real-Time PCR Experiments) guidelines, the amplification efficiencies (E) of reported runs were between 97% and 113% and *R*^2^ > 0.9 with minimum two technical replicates per reaction. The Bio-Rad SYBR Green Assays used were Nestin (qHsaCED0044457), Tuj1 (qHsaCED0005794), Olig2 (qHsaCED0007834), Gfap (qHsaCID0022307), BDNF (qHsaCED0047199), and Gapdh (qHsaCED0038674). Relative expression data were normalized to the reference gene Gapdh to control for variability in expression levels and were analyzed using the Livak and Schmittgen (i.e., 2^−ΔΔCT^) and Pfaffl methods. The relative expression of each target was assessed by unpaired two-tailed *t* test. A *p* value of less than 0.05 was considered significant.

### Enzyme-Linked Immunosorbent Assay (ELISA)

Brain-derived neurotrophic factor (BDNF) released into the conditioned medium of drNPC cultures that were differentiated towards a neural lineage was measured by antigen-capture ELISA at different time points and compared to the release of BDNF in the conditioned medium of mature neurons (cat #1520, ScienCell). Conditioned medium from each group was collected, centrifuged, and then stored at − 80 °C until assaying. BDNF concentrations were measured by ELISA kit (BDNF E_max_ Immunoassay System, Promega Corporation, USA), according to the manufacturer’s instructions. Briefly, 96-well ELISA immunoplates were coated with Anti-BDNF (CatNb#G700B) diluted 1/1000 in carbonate buffer (pH 9.7), and incubated at 4 °C overnight. The following day, all wells were washed with TBS-Tween 0.5% before incubation with Block/Sample buffer 1× at room temperature for 1 h without shaking. After blocking, standards and samples were added to the plates and incubated and shaken (450 ± 100 rpm) for 2 h at room temperature. Subsequently, after washing with TBS-Tween wash buffer, plates were incubated for 2 h with Anti-Human BDNF (1:500 dilution in Block & Sample 1× Buffer) at 4 °C. After incubation, plates were washed five times with TBS-Tween 0.5% wash buffer and 100 μl of diluted Anti-IgYHRP Conjugate was added to each well (1:200 dilution in Block & Sample 1X Buffer) and incubated for 1 h at room temperature with shaking (450 ± 100 rpm). Then, plates were washed five times with TBS-Tween 0.5% wash buffer and 100 μl of TMB One Solution was added to each well. Following 10 min incubation at room temperature with shaking (450 ± 100 rpm) for the BDNF plate, a blue color formed in the wells. After stopping the reaction by adding 100 μl of 1 N hydrochloric acid, the absorbance was read at 450 nm on a microplate reader (Synergy 4) within 30 min of stopping the reactions. Concentration of released BDNF in the supernatants was determined according to the standard curves. BDNF concentrations were compared using an unpaired, two-tailed *t* test for each time point.

### Behavioral Tests

Behavioral analysis was performed using the foot fault task, measuring gross motor functions such as coordination and balance, as well as fine sensorimotor function like reaching and stepping [33], 3 days prior to injury (baseline) and at 3, 8, 18, and 32 days post-stroke; and the cylinder test at 3 days prior to injury and at 3 and 32 days post-stroke. Detailed methods of behavioral tests can be found in the supplementary material. All behavioral tests were recorded with a digital camera (SX 60 HS, Canon) and viewed on VLC Media Player (Version 2.2.1, VideoLAN Organizarion). Videos were scored by a blinded observer.

### Statistical Analysis

Statistical analysis was performed using Prism 6 (GraphPad Software, San Diego, CA) and IBM SPSS Statistics (International Business Machines Corp., Armonk, NY). Data was analyzed using a variety of statistical methods which can be found in the supplementary material. All data is reported as mean ± SEM.

## Results

### drNPCs Are Neurally Committed at the Time of Transplant

**Fig. 1:**
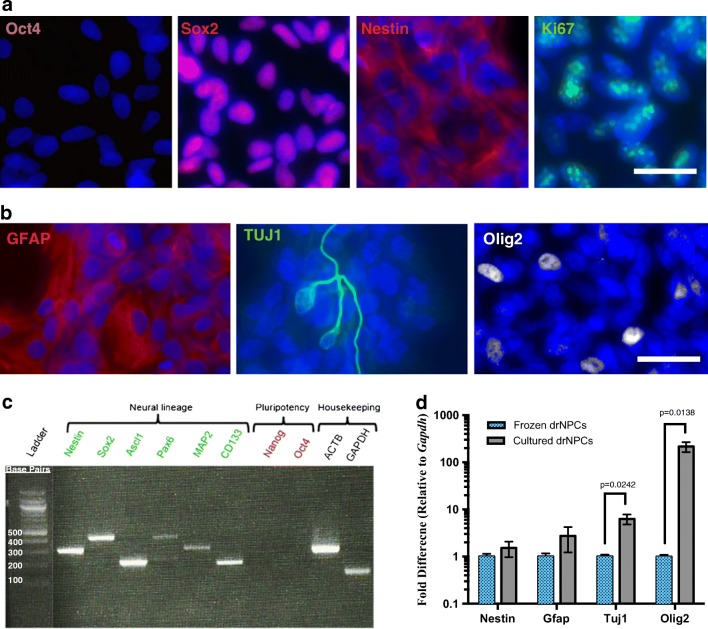
drNPCs remain in a neural precursor state in vitro at time of transplantation. **a** Immunocytochemistry reveals that drNPCs do not express the pluripotency marker Oct4, but do ubiquitously express neural precursor cell markers Sox2 and Nestin, while 71.8 ± 4.0% of cells express the proliferation marker Ki67. **b** Cultured drNPCs can also express differentiated neural cell markers for astrocytes (GFAP), neurons (TUJ1), and oligodendrocytes (Olig2). **c** Polymerase chain reaction confirms that drNPCs express neural markers Nestin, Sox2, Ascl1, Pax6, MAP2, CD311, and do not express pluripotency markers Nanog and Oct4. **d** Cultured drNPCs express equivalent levels of Nestin and Gfap, but increased Tuj1 and Olig2 mRNA compared to the frozen drNPCs. Data are shown as mean ± SEM. Gene expression levels are relative to frozen drNPCs and normalized to the reference gene Gapdh. *n* = 3/cohort. **a**, **b***n* = 3, with three technical replicates per stain. Scale bar = 25 μm, blue = nuclear stain

To confirm the identity of drNPCs, we characterized their expression of pluripotency and neural markers using immunostaining and polymerase chain reaction (PCR). We examined Oct4 expression as a measure of pluripotency; Sox2 and Nestin for NPCs; Ki67 to assess proliferation; GFAP for astrocytes; TUJ1 for neurons; and Olig2 for oligodendrocytes. We found that drNPCs do not express Oct4, and ubiquitously express NPC markers Sox2 and Nestin, indicating a precursor phenotype (Fig. 1a, Supplementary Fig. 1). In addition, we determined that 71.8 ± 4.0% of drNPCs were Ki67^+^ in vitro (Fig. 1a). We also confirmed that drNPCs have the ability to differentiate into the three neural subtypes and express GFAP, TUJ1, and Olig2 (Fig. 1b). PCR analysis confirmed the expression of neural lineage markers including Nestin, Sox2, Ascl1, Pax6, MAP2, and CD133 and the absence of pluripotency markers Nanog and Oct4 (Fig. 1c). RT-qPCR analysis comparing the expression levels of neural markers Nestin, Tuj1, Olig2, and Gfap in drNPCs prior to culturing (from frozen vials) and drNPCs that were cultured prior to transplantation (cultured drNPCs) revealed similar expression of Nestin and Gfap between the two cell populations and increased expression of Tuj1 and Olig2 in cultured drNPCs (Fig. 1d). Collectively, these results confirm that drNPCs are neurally committed, remain in a precursor state at time of transplant, and can give rise to all three neural cell types.

### Cell Viability Is Not Dependent on the Transplant Vehicle

**Fig. 2:**
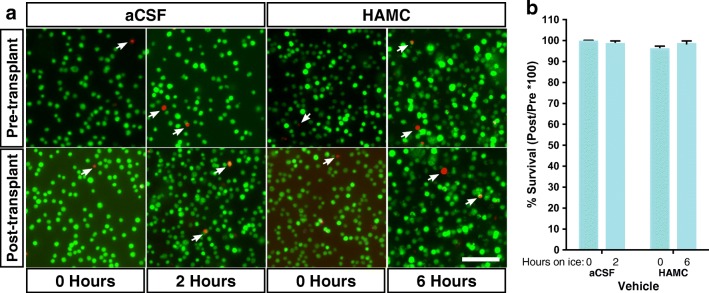
drNPC transplant injection paradigm results in minimal cell death. **a** Live/dead assay images before and after injection at the first and last injection time points within each vehicle. All cells were maintained on ice within their respective vehicle until transplanted. **b** There is no significant difference in % survival between groups at any time point. Cell survival as a result of injection through the syringe was greater than 95% in all instances (aCSF 0 h = 99.60 ± 0.53%, 2 h = 98.52 ± 1.29%; HAMC 0 h = 96.00 ± 1.32%, 6 h = 98.49 ± 1.32%). Data presented as mean ± SEM; *n =* 3 per vehicle within each time point, with three technical replicates each; green cells = live cells, red cells (white arrowheads) = dead cells; scale bar = 100 μm

With the goal of enhancing cell viability at the time of transplant and within the host, we tested two transplant vehicles; (1) artificial cerebrospinal fluid (aCSF), a buffer to mimic circulating CSF, and (2) a hyaluronan methylcellulose hydrogel (HAMC), which has been shown to improve xenograft survival in the CNS [17, 22]. To determine the effect of vehicle on the number of live cells at the time of transplantation, we performed a live/dead assay on cells prior to and following injection through the syringe. Cell viability was determined at 0 h and 2 h (aCSF) or 6 h (HAMC) after cell preparation (Fig. 2a), which was reflective of the longest elapsed time from preparation to transplanting in vivo. Cell viability was always > 88% of the original cell population (aCSF 0 h = 95.5 ± 0.2%, 2 h = 94.5 ± 1.5%; HAMC 0 h = 95.2 ± 0.8%, 6 h = 88.2 ± 0.3%). Cell viability following injection through the syringe, relative to the numbers of viable cells placed in the syringe, was > 95% in all conditions and was not significantly different between vehicles at any time point (Fig. 2b). Thus, all mice received a minimum of ~ 85,000 viable drNPCs at the time of transplantation.

### drNPC Transplantation Promotes Functional Recovery

**Fig. 3:**
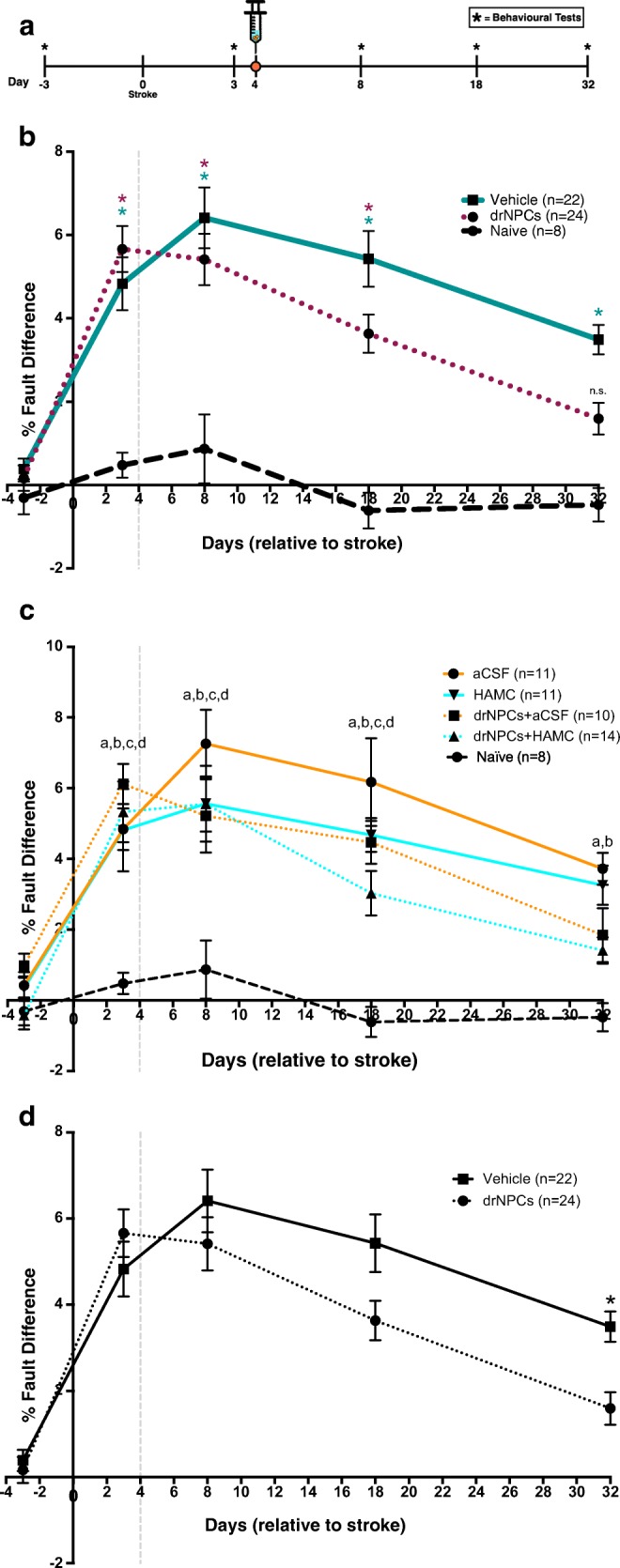
drNPC transplantation promotes functional recovery. **a** Mice were tested for functional performance on the foot fault test at 3 days prior to stroke and 3, 8, 18, and 32 days post-stroke, and sacrificed 32 days post-stroke, when tissue analysis was performed. **b**–**d** All stroke-injured mice have significant functional deficits by 3 days following stroke. **b** drNPC transplants promote functional recovery back to uninjured control levels by 32 days post-stroke, whereas vehicle-only injections did not. **c** The transplant vehicle had no impact on functional recovery, as only those mice that received drNPCs recovered to naïve levels and those that received vehicle injections did not, irrespective of transplant vehicle (HAMC or aCSF). **d** Mice that received drNPCs had significantly better performance on the foot fault test than those that received vehicle-only injections at 32 days post-stroke. Data is presented as mean ± SEM; * = **b** significantly different from naïve **d** significant difference between groups; **c** a (aCSF alone), b (HAMC alone), c (drNPCs + aCSF), d (drNPCs + HAMC) = significantly different from naïve; n.s. = not significant, b) *p* < 0.0001, c) *p* < 0.003, and d) *p* < 0.05

To determine the efficacy of drNPCs for stroke recovery, we used a clinically relevant model of ET-1 stroke in the sensorimotor cortex. This resulted in consistent tissue damage, gliosis, and functional impairment in the foot fault task as early as 4 days post-stroke, which was maintained up to 32 days post-stroke, the longest time point examined (Supplementary Fig. 2). Cultured drNPCs were injected directly into the stroke lesion at 4 days post-stroke based on previous work [17]. Mice were tested in the foot fault task at 3 days prior to stroke to establish a baseline measure, and at 3, 8, 18, and 32 days post-stroke (Fig. 3a). Only stroke-injured mice that had significant motor impairments on the foot fault task by 8 days post-stroke (deficits present at day 3 or day 8) were included in our analysis (aCSF = 11/12 mice; HAMC = 11/13 mice; drNPCs+aCSF = 10/13 mice; drNPCs+HAMC = 14/15 mice). Animals not showing any deficits on either day 3 or 8 post-stroke were excluded from our analysis and completely removed from the study. These exclusion criteria were used to prevent animals that did not actually have a deficit following stroke from skewing the outcomes of the study to falsely showing improved performance.

Stroke-injured mice that received vehicle-only (aCSF or HAMC) injections displayed functional impairments at all times examined, relative to their own baseline performance as well as compared to naïve (uninjured) controls (Fig. 3b). Interestingly, mice that received drNPC transplants in either HAMC or aCSF recovered to their baseline performance by 32 days post-stroke and were not significantly different from naïve performance (Fig. 3b). Our analysis revealed that the observed functional recovery was due to drNPC transplants, irrespective of vehicle (Fig. 3c). We investigated this relationship further by comparing the performance of mice that received drNPCs vs vehicle-alone injections and found that mice that received drNPCs performed significantly better than those that received vehicle-only injections at day 32 post-stroke (Fig. 3d). Functional recovery following drNPC transplantation was seen in both male and female mice (Supplementary Fig. 3a), indicating that drNPC transplants promote recovery regardless of recipient sex.

To further assess functional outcomes, we performed the cylinder test in a subset of mice that demonstrated impairments in the foot fault task post-stroke. Similar to what we observed in the foot fault task, only mice that received drNPCs recovered back to baseline levels, whereas mice that received vehicle-only injections did not recover (Supplementary Fig. 2b). Taken together, these data support the conclusion that drNPC transplants promote functional recovery.

### Long-Term Surviving drNPCs Are Found in a Subpopulation of Stroke-Injured Mice

**Fig. 4:**
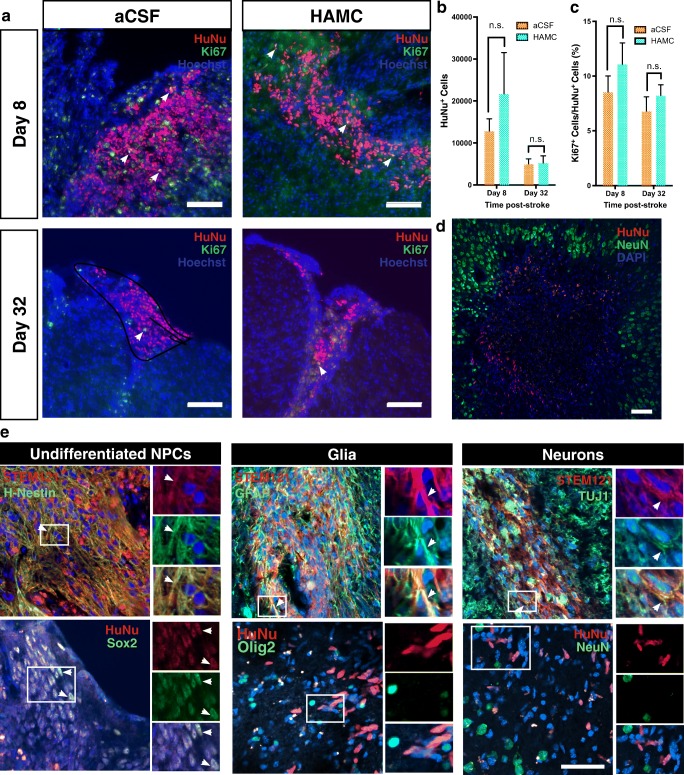
Transplanted drNPCs can survive and proliferate in the stroke-injured cortex. **a** HuNU^+^ (red) drNPCs are seen within the stroke-injured cortex of SCID/Beige mice at 8 and 32 days post-stroke. A subpopulation of HuNu^+^ cells are Ki67^+^ (green) at both survival times. **b** The number of HuNu^+^ drNPCs found within the stroke-injured cortex at 8 or 32 days post-stroke was not significantly different between cells transplanted in aCSF or HAMC (8 days, *p* = 0.34; 32 days*, p* = 0.99). Significantly fewer HuNu^+^ cells were observed between 8 and 32 days post-stroke when transplanted in HAMC (*p =* 0.019) but not aCSF (*p* = 0.34). **c** There was no significant difference between transplant vehicles in the percentage of Ki67^+^/HuNu^+^ drNPCs at 8 or 32 days post-stroke (day 8, *p* = 0.49; day *32, p* = 0.79). There was also no significant difference in Ki67^+^/HuNu^+^ drNPCs between 8 and 32 days within each vehicle group (aCSF, *p* = 0.66; HAMC, *p* = 0.46). Percent of ki67^+^/HuNu^+^ drNPCs at Day 8, 8.5% ± 1.5 (aCSF) and 11.0% ± 2.0 (HAMC); and Day 32, 6.8% ± 1.3 (aCSF) and 8.2% ± 1.0 (HAMC). **d** IHC reveals that drNPCs (HuNu^+^) remain at the boundary of the stroke injury (demarcated by NeuN^+^ cells) following transplantation into the stroke-injured cortex 4 days post-stroke. **e** Surviving drNPCs at 32 days post-stroke mostly remain undifferentiated (Sox2, Nestin). The majority of differentiated drNPCs primarily gave rise to astrocytes (GFAP), while a smaller population gave rise to immature neurons (TUJ1). No drNPCs differentiated to mature neurons (NeuN) or oligodendrocytes (Olig2). Data are represented as mean ± SEM, **a** Arrowheads indicate Ki67^+^/HuNu^+^ cells. **e** Arrowheads indicate colocalization between markers. **a**, **d** Scale bars = 100 μm **(b)**, *n* = 4 for both vehicles at day 8 and *n* = 7 for both vehicles at day 32. **c***n* = 4 for both vehicles at day 8, and *n* = 6 for aCSF and *n* = 3 for HAMC at day 32 **(e)** Scale bar = 50 μm, at least three brains were analyzed per marker and representative images were used

To assess drNPC survival post-transplant, we sacrificed mice at 8 and 32 days post-stroke and stained for human cell markers HuNu and/or STEM121. At 8 days post-stroke (i.e., 4 days post-transplant), all mice (100%) had drNPCs at the site of injection. By 32 days post-stroke (i.e., 28 days post-transplant), the time when functional recovery was observed, drNPCs were only observed in 71% (HAMC, 8/14; aCSF, 9/10) of transplanted brains. A Fisher’s exact test and chi-square test revealed no significant association between vehicle and cell survival (*p* > 0.05)*.* Interestingly, in all brains that had drNPCs present, irrespective of the time of sacrifice, transplanted cells were confined within the lesion boundary demarcated by NeuN expression, and did not penetrate deep into the uninjured tissue (Fig. 4d).

The total number of viable HuNu^+^ drNPCs within the transplanted brains 8 days post-stroke was 12,780 ± 2963 when delivered in aCSF and 21,633 ± 9880 when delivered with HAMC (Fig. 4a, b). A decline in the numbers of surviving HuNu^+^ cells was observed by day 28 post-transplantation in both vehicles; by 32 days post-stroke, the total numbers of HuNu^+^ cells were 4961 ± 1266 for cells transplanted in aCSF and 5130 ± 1815 for cells transplanted in HAMC (Fig. 4b). However, this decrease was only significant for brains that received cells in HAMC. No significant difference in drNPC survival was observed between vehicles at 8 or 32 days following stroke (Fig. 4b).

Proliferation of drNPCs at 8 and 32 days post-stroke was measured by counting the number of Ki67^+^/HuNu^+^ cells as a percent of all HuNu^+^ cells in brains (Fig. 4a). We found no significant difference between the two times examined in either vehicle and no significant effect of transplant vehicle (Fig. 4c).

We further characterized the in vivo profile of surviving drNPCs (HuNu^+^ or STEM121^+^ cells) at 32 days post-stroke using immunostaining for Sox2 and Nestin (undifferentiated NPCs), GFAP (astrocytes), TUJ1 (immature neurons), NeuN (mature neurons), and Olig2 (oligodendrocytes). Irrespective of the transplant vehicle, the vast majority of surviving drNPCs remain undifferentiated (Sox2^+^, Nestin^+^). A subpopulation of drNPCs expressed GFAP and a small minority expressed the immature neuronal marker TUJ1, but no mature neurons (NeuN^+^) or oligodendrocytes (Olig2^+^) were observed at 28 days post-transplant (Fig. 4e).

### drNPC Transplant Survival Is Not Necessary for Recovery

**Fig. 5:**
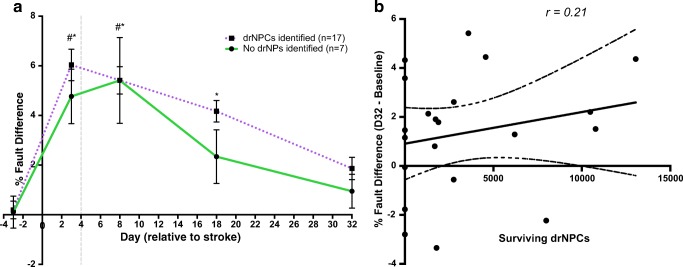
Xenograft survival is not necessary for functional recovery. **a** Performance in the foot fault task revealed no significant difference in functional recovery between mice that had drNPCs present at day 32 versus mice with no drNPCs present. **b** There is no significant correlation (*r* = 0.21, *n* = 21, *p =* 0.36) between the absolute drNPC survival numbers in vivo and functional recovery by 32 days post-stroke. a* **=** brains with surviving drNPCs different from baseline, # *=* brains with no drNPC survival different from baseline; *p* < 0.05

Approximately 30% of brains that received drNPC transplants did not have drNPCs present at 28 days post-transplant when functional recovery was observed. We asked whether cell survival was necessary for maintaining functional recovery by comparing behavioral performance in mice with (*n* = 17) and without (*n* = 7) drNPCs present at day 32 post-stroke. Our findings reveal that functional recovery occurs irrespective of the presence of cells at day 32 post-stroke (Fig. 5a). Interestingly, mice without cells at 32 days post-stroke had already recovered by 18 days post-stroke. There was no significant difference in performance between the two groups at any time-point tested. Moreover, Pearson’s and Point-Biseral analyses revealed a significant correlation between functional performance at day 32 (compared to baseline) and the injection of drNPCs into the cortex (*r* = 0.37, *n* = 46, *p* = 0.011), but no correlation between whether the drNPCs were present on day 32 post-stroke (r = − 0.17, *n* = 24, *p* = 0.43) or with the absolute number of surviving drNPCs (*r* = 0.21, *n* = 21, *p* = 0.36) (Fig. 5b). These findings reveal that long-term transplanted cell survival is not necessary for maintaining functional recovery.

### Lesion Volume and Glial Scarring Are Unaffected by Transplant and Not Correlated to Functional Recovery

**Fig. 6:**
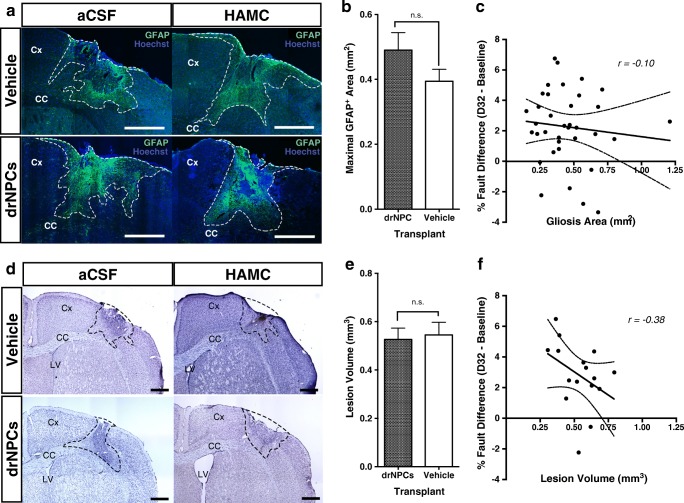
Glial scarring and lesion volume are unaffected by treatment and do not correlate with functional recovery. **a** Gliosis was present in brains from all groups at 32 days post-stroke. **b** There was no significant difference in terms of maximal GFAP^+^ area between drNPC transplanted brains and vehicle-only brains (*n =* 20, drNPC transplant; *n* = 16, vehicle-only) **c** There is no significant correlation (*r* = − 0.10, *n* = 36, *p =* 0.55) between the extent of gliosis and the functional performance at 32 days post-stroke compared to baseline performance. **d** Cresyl violet staining reveals stroke lesions in all groups at 32 days post-stroke. **e** No significant difference in lesion volume is seen between drNPC transplant and vehicle-only treated brains (*n =* 8 per group)*.***f** There is no significant correlation *(r* = − 0.38, *n* = 16, *p* = 0.15) between the lesion volume and the functional performance at 32 days post-stroke compared to baseline performance. Dashed line = **a** GFAP^+^ expression boundary or **d** cresyl violet lesion boundary, dashed line within graphs. **c**, **f** *=* 95% confidence bands, Cx = cortex, CC = corpus callosum, n.s. = not significant, *r* = Pearson’s coefficient, significance based on *p* < 0.05. Data are represented as mean ± SEM. Scale bars = 500 μm

We asked whether drNPC transplants affected the extent of gliosis and the size of the lesion following ET-1 stroke, and whether these outcomes were related to the observed motor recovery. The extent of gliosis was determined using GFAP staining in vehicle and drNPC transplanted mice at 32 days post-stroke (Fig. 6a) by measuring the maximal cortical GFAP^+^ area, which was strongly correlated (*r = 0.89, n = 35, p* < 0.001) to total gliosis volume per brain (Supplementary Fig. 4). A comparison between the gliotic response in mice that received drNPCs versus vehicle-only injections (*n* ≥ 16 per group) revealed no significant difference between groups (Fig. 6b). There was also no significant difference between vehicle type in drNPC or vehicle-only treated groups (Supplementary Fig. 5a). Furthermore, a Pearson’s correlation revealed no significant correlation (*r =* − 0.102, *n* = 36, *p =* 0.554) between the size of the glial scar and functional performance at 32 days post-stroke (Fig. 6c).

A similar observation was made comparing the ischemic lesion volume between vehicle-only and drNPC-transplanted mice. Cresyl violet staining revealed no difference in the size of the lesion between mice that received vehicle only versus drNPCs (Fig. 6d, e) or between the vehicle type within the groups (Supplementary Fig. 5b). A Pearson’s correlation also revealed no significant correlation (*r* = − 0.38, *n* = 16, *p* = 0.15) between behavioral recovery and lesion volumes (Fig. 6f). Hence, drNPC transplants do not affect the extent of gliosis or size of the lesion volume post-ET-1 stroke and these tissue outcomes are not correlated with functional improvement.

### drNPC Transplants Increase Perilesional Expression of Synaptophysin

**Fig. 7:**
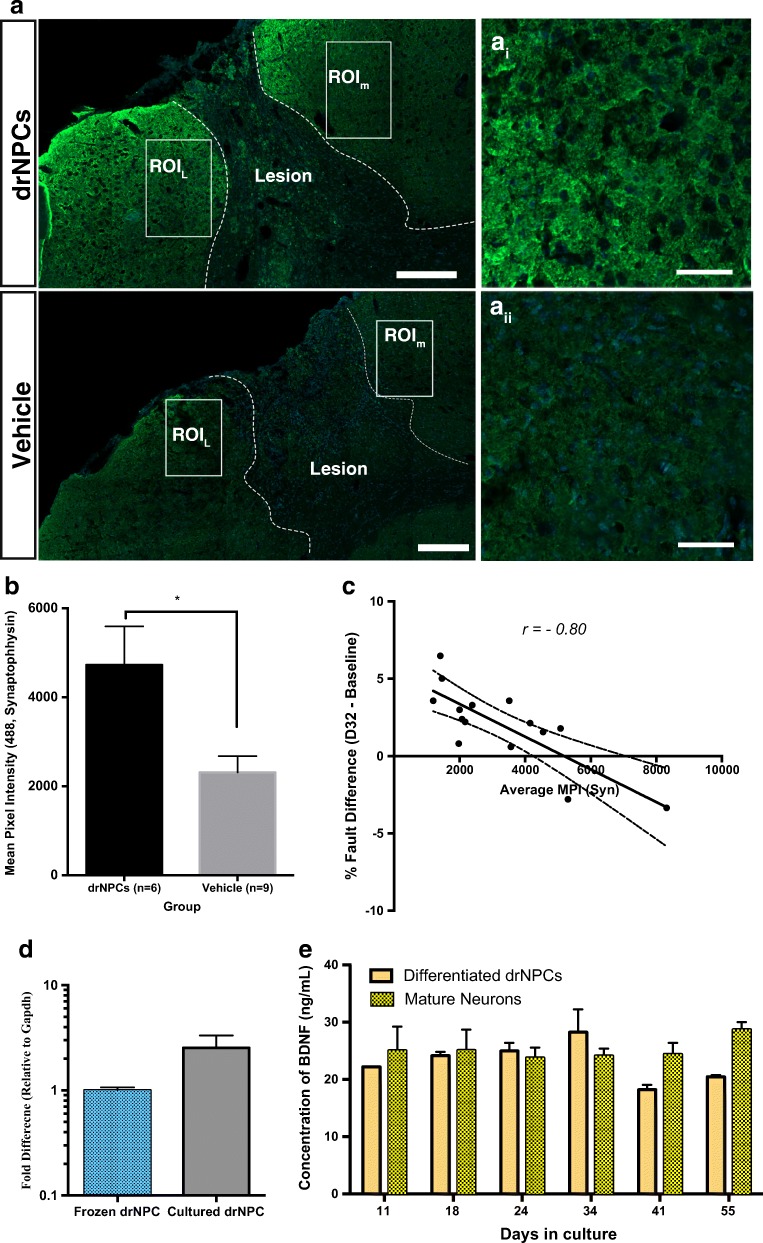
drNPC transplants result in increased synaptophysin expression in the perilesional area. **a** Two ROIs within the perilesional tissue (medial and lateral) were selected in one coronal section per brain analyzed. The ROIs were imaged through eight optical planes and the settings were all kept identical for each section. ai–ii: higher magnification images of perilesional areas. **b** Mice that received drNPCs had significantly greater MPI for Alexa Fluor 488 Staining (Synaptophysin) within the ROIs than mice that received vehicle alone injections. **c** Pearson correlation analysis reveals that synaptophysin MPI is strongly correlated (*r* = − 0.80, *n =* 15, *p* = 0.0003) with improved functional outcomes. **d** RT-qPCR analysis of drNPCs. BDNF expression levels are relative to frozen drNPCs and normalized to the reference gene Gapdh. Both frozen and cultured drNPCs express BDNF. Data are shown as mean ± SEM. *n* = 3/cohort. **e** Differentiated drNPCs release BDNF at levels comparable to that of mature neurons in vitro, as determined by quantification of BDNF (ng/mL) release using antigen-capture. The populations of cells tested were mature neurons (positive control) and drNPCs in differentiation conditions. Data are shown as mean ± SEM. *n* = 3 independent samples per timepoint*.* Dashed lines = lesion boundary, white boxes = ROIs ROI_m_ = Medial ROI, ROI_L_ = Lateral ROI, **a** scale bar = 200 μm, a_i–ii_ scale bar = 50 μm, **b***n* = 6 for drNPC group and *n* = 9 for vehicle group, * = significant difference; *p* = 0.012

To investigate the possibility that drNPC transplants influenced recovery by promoting synaptic plasticity, we examined the expression of synaptophysin, a presynaptic vesicle protein, in treated stroke-injured brains. Using immunohistochemistry and confocal imaging (Fig. 7a), we compared the mean pixel intensity (MPI) of synaptophysin expression in the perilesional tissue of stroke-injured mice that received drNPCs (*n* = 6) and those that received vehicle-only injections (*n* = 9). Mice that received drNPC transplants had a significant increase (*p* = 0.012) in synaptophysin expression compared to vehicle-treated mice (Fig. 7b). A Pearson’s correlation also revealed that decreased functional impairment is strongly correlated *(r =* − 0.80, *n* = 15, *p* = 0.0003) with increased synaptophysin MPI (Fig. 7c). This supports the hypothesis that drNPC transplants lead to increased synaptic plasticity which may underlie the observed functional recovery.

To elucidate a potential mechanism by which drNPC transplants exert their beneficial effects, we asked whether drNPCs express and secrete brain-derived neurotrophic factor (BDNF), which is known to promote neuroplasticity [34]. RT-qPCR analysis revealed that BDNF is expressed in both frozen and cultured drNPCs (Fig. 7d). An ELISA analysis of culture medium derived from drNPC cultures that were differentiated towards a neural lineage over time reveals that BDNF is released at levels comparable to mature neurons (Fig. 7e). Hence, BDNF-mediated plasticity may play a role in the functional recovery observed following drNPC transplantation.

## Discussion

We have shown that drNPC transplantation during the subacute phase in a pre-clinical mouse model of stroke is able to promote functional recovery, regardless of the transplant vehicle or the sex of the recipient. Furthermore, we found that functional recovery does not require the long-term survival of transplanted cells, and that recovery is maintained beyond transplant survival. In the brains of mice that did have surviving drNPCs at late survival times, the majority of the transplanted drNPCs remained undifferentiated and non-proliferative. Most interesting, brains that received drNPC transplants had higher levels of synaptophysin in the perilesional stroke-injured cortex, supporting the idea that synaptogenesis may underlie the drNPC-mediated recovery.

Cell survival is a challenge common to transplant therapies in general. Herein, we used two transplant vehicles with the goal of establishing the best parameters to enhance cell survival and promote recovery. Interestingly, the frequency and absolute number of viable drNPCs observed in vivo were not different between HAMC and aCSF. Previous studies report that HAMC has pro-survival properties and improves cell transplant survival outcomes using murine cells [17, 35–37], which has been attributed in part to the immunomodulatory effects of HAMC [38–41]. Accordingly, the lack of pro-survival effects of HAMC in this study may be due to the immunomodulatory advantage of HAMC being negated in the immunodeficient mouse strain (lacking adaptive immune cells). Of note, drNPC proliferation was also not affected by the vehicle. In both vehicles, the proliferative ability of drNPCs decreased following injection into the stroke-injured brain; dropping from 71.8 ± 4.0% at time of transplantation to approximately 10% by 4 days post-transplant. Importantly, we also found no evidence of tumor formation in any of the animals, similar to previous work with drNPCs [42].

Our results indicate that the long-term survival of transplanted cells is not necessary for maintaining functional recovery, although their presence at early times is important, as vehicle-only treated mice did not recover. We found no correlation between functional recovery and the extent of gliosis or lesion volumes, consistent with observations in other models of stroke where interventions lead to recovery but had no effect on tissue outcomes [43–45]. The mechanism by which transplanted cells mediate recovery is still unknown but there is evidence that suggests transplanted cells can promote recovery through trophic support, by promoting plasticity and synaptogenesis, inducing angiogenesis, immunomodulation, reducing excitotoxicity, and even activating endogenous cells to proliferate and migrate to the site of the lesion [15, 16, 18, 20, 43, 45–55]. Notably, the short-term survival of the transplanted cells is consistent with the hypothesis that the presence of drNPCs promotes recovery through an indirect mechanism.

Our observation that drNPC transplants lead to functional recovery and increased synaptophysin expression in the perilesional stroked hemisphere suggests that one underlying mechanism for drNPC-mediated recovery for stroke is enhancing host brain plasticity; through increased synaptogenesis via the development of new synaptic junctions, potentially resulting from axonal sprouting and endogenous cortical remapping [43, 56]. Exploring the secretome of transplanted drNPCs may provide further insight into the mechanisms and pathways that result in functional recovery.

Supplementary to our findings, recent studies transplanting drNPCs that were pre-differentiated towards an oligodendrogenic fate prior to transplantation in a rat model of spinal cord injury resulted in improved functional outcomes via migration and integration within the injured tissue, where they participated in tissue sparing and axonal remyelination [42]. Thus, it is possible that the observed mechanism of recovery depends on a variety of factors, such as injury, host, and status of drNPCs, which is an important consideration for drNPCs as autologous transplants since they could have additional mechanisms of action related to cell replacement in humans.

drNPCs have the potential to provide a safe, autologous, and plentiful source of cells for clinical neural repair strategies and our findings support the conclusion that drNPCs are a promising candidate to treat stroke. The potential of translating the results from our study to the clinic raises important questions with regard to the optimal timing of transplantation and the associated mechanism that induces recovery. Transplantation of drNPCs in a different model that produces a larger lesion or a chronic model of stroke, in addition to selective ablation of transplanted cells at various times post-stroke, may provide additional insight into the optimal therapeutic window for transplantation and further our understanding of the underlying cell-based mechanisms that promote recovery. Further understanding of these mechanisms will support the development of novel therapeutics for neural repair.

## Electronic Supplementary Material

ESM 1 (DOCX 4560 kb)
