# Supplementary material for: Transplantation of Directly Reprogrammed Human Neural Precursor Cells Following Stroke Promotes Synaptogenesis and Functional Recovery
Source: Transl Stroke Res. 2019 Feb 12;11(1):93–107. doi: 10.1007/s12975-019-0691-x (PMC6957566; doi:10.1007/s12975-019-0691-x)
Supplement: Supplementary file 1 — (DOCX 4560 kb) [file 12975_2019_691_MOESM1_ESM.docx]

Transplantation of directly reprogrammed human neural precursor cells following stroke promotes synaptogenesis and functional recovery

**Authors:** Ilan Vonderwalde^1^†, Ashkan Azimi^2^†, Gabrielle Rolvink^3^, Jan-Eric Ahlfors^4^, Molly S. Shoichet^1^, Cindi M. Morshead^1,2,3*^

**Affiliations:**

^1^ Institute of Biomaterials and Biomedical Engineering, University of Toronto, Toronto, Ontario, M5S 3E1, Canada.

^2^ Institute of Medical Sciences, University of Toronto, Toronto, Ontario, M5S 3E1, Canada.

^3^ Department of Surgery, Division of Anatomy, University of Toronto, Toronto, Ontario, M5S 3E1, Canada.

^4^ New World Laboratories, Laval, Quebec, H7V 5B7, Canada.

†Authors contributed equally to the work

***Address correspondence to:**

Cindi Morshead, PhD

Department of Surgery, Donnelly Centre, University of Toronto, Ontario, M5S 3E1, Canada

Phone : 1-416-946-5575

FAX : 1-416-946-5545

E-mail : [cindi.morshead@utoronto.ca](mailto:cindi.morshead@utoronto.ca)

**Supplementary Methods:**

*Inclusion Criteria for Animals*

Outliers were removed by robust regression and outlier removal (ROUT) method on Prism GraphPad (Q=1.0%) based on foot fault performance following stroke and prior to treatment. The mean and standard deviation (STDEV) for baseline performance was calculated for all mice receiving a stroke. Mice that did not show a deficit in % Fault Difference greater than 2 STDEV from the baseline mean up to 8 days following stroke were removed from the study (1 outlier was removed and 6 animals were excluded because they did not exhibit a functional deficit).

*Reprogramming somatic bone marrow cells to drNPCs*

Bone marrow somatic cells (Lonza, Walkersville, MD) were centrifuged and resuspended in StemPro^®^ MSC SFM CTS^TM^ complete medium (Invitrogen) and cultured for 1 week. Cells were then collected and directly reprogrammed into NPCs by transient nucleofection (4D Nucleaofector^TM^, Lonza) with a synthesized polycistronic vector containing human musashi-1 (Msi1), neurogenin-2 (Ngn2), and methyl-CpG binding domain protein 2 (MBD2). Reprogrammed cells were cultured in low oxygen conditions (5% CO_2_; 5% O_2_ and 37°C) in Human NeuroCult^TM^-XF proliferation medium (StemCell Technologies) supplemented with epidermal growth factor (EGF) [20 ng/ml] (CellGenix), fibroblast growth factor-2 (FGF-2) [30 ng/ml] (CellGenix), Valproic Acid (VPA) [1 mM] (Sigma-Aldrich), and Noggin [20 ng/ml] (R&D Systems). Following 2 days in culture, the synthetic plasmid containing Msi1, Ngn2, and MBD2 was re-introduced to the cells through lipofection (Lipofectamine^®^ LTX & Plus^TM^ Reagent, Invitrogen). After 6 days in culture VPA and Noggin were replaced by heparin [100 ng/ml] (Scientific Protein Laboratories).

*Maintenance and expansion of drNPCs*

Human drNPCs were cultured and expanded on Corning^®^ CellBIND^®^ culture dishes (Corning, Product #2394 to #3296, Tewksbury, MA) as monolayers in low oxygen conditions (at 5% CO_2_; 5% O_2_ and 37°C) in Human NeuroCult^TM^-XF medium (StemCell Technologies) supplemented with EGF [20 ng/ml] (Peprotech), FGF-2 [30 ng/ml] (Peprotech), and heparin [100 μg/ml] (Scientific Protein Laboratories). Cells were detached using Accutase (Innovative Cell, Technologies, Inc. Cat #AT-104) and either passaged or used for transplants once they reached ~ 80% confluency ( 4–7 days of culturing). Cell feeding occurred every 36 hours by replacing 50% of the media.

*HAMC preparation*

The HAMC hydrogel was prepared as previously described (Ballios et al., 2015) using a blend of hyaluronan (HA, 1400-1800 kDA, Novamatrix, Drammen, Noraway) and methylcellulose (MC, 300 kDa, Shin-Etsu, Tokyo Japan). Sterile-filtred HA and MC were dissolved in artificial cerebrospinal fluid (aCSF; 126 mM NaCl, 3 mM KCL, 26 mM NaHCO_3_, 1.25 mM NaH_2_PO_4_, 10 mM D-Glucose, 2 mM MgCl_2_, and 10 mM CaCl_2_ dissolved in double distilled H_2_O) at a concentration of 1% HA (w/v) and 1% MC (w/v) producing a final solution of 1/1 HAMC. The 1/1 HAMC was mixed in a SpeedMixer (DAC 150 FVZ, Siemens) for 30 seconds at 3,500 RPM, centrifuged for 1.5 minutes, and placed on a shaker at 4˚C for 14 hours. The following day, the HAMC hydrogel was mixed again for 30 seconds at 3,500 RPM, centrifuged for 5 minutes, and stored on ice for 20 minutes, repeating until no bubbles remained in the hydrogel solution. drNPCs in aCSF were added to the 1/1 HAMC to achieve a final concentration of 0.75/0.75%wt HAMC.

*Detailed Cell Transplantation*

A 1µL cell suspension (100,000 cells) was injected into the same location as the ET-1 injection 4 days prior at a rate of 0.1µL/min using a 2.5µL Hamilton Syringe with a 26 gauge, .375” long needle (Hamilton, Reno, NV). The syringe was removed 10 minutes after the injection to prevent backflow. Transplants using aCSF were carried out within 2 hours of cell suspension, whereas transplants using HAMC were carried out within 6 hours of cell suspension.

*Tissue and cell processing*

Mice received an overdose of Avertin (Sigma Aldrich) and were transcardially perfused with cold 0.01M phosphate buffered saline (PBS) solution followed by cold 4% paraformaldehyde (PFA) solution (pH=7.4). Brains were removed and placed in 4% PFA for 3–6 hours followed by 20% sucrose in 0.01M PBS solution overnight. Brains were cryosectioned at 20µm along the coronal plane and collected in series on Superfrost Plus Microscope Slides (Cat #12-550-15, Fisherbrand).

For cell work, *in vitro* sister cell cultures of drNPCs were fixed at the same time cells were collected for transplants. Cultures were fixed using 4% PFA solution (pH=7.4) for 20 minutes and washed in PBS.

*Cresyl violet staining*

Slides were dehydrated by placing in 100% EtOH (2 min), 100% EtOH (2 min), 95% EtOH (2 min), 95% EtOH (2min), 70% EtOH (2 min) and then ddH_2_O (1 min) followed by 0.25% cresyl violet solution for up to 15 minutes. Slides were rinsed in ddH_2_O then 0.25 acetic acid solution (dissolved in 100% EtOH), and placed in 95% EtOH (2 min), 100% EtOH (2 min), and xylene (2 min). Slides were then mounted with coverslips using a xylene-based medium (Cytoseal^TM^ XYL, ThermoFisher Scientific).

*Synaptophysin immunostaining and analysis*

To stain, sections were permeabilized for 20 minutes using 1% TritonX solution, followed by a 1 hour block in 5% Bovine Serum Albumin (BSA) in 0.03% TritonX solution. Following staining with primary and secondary antibodies (as previously described), sections were imaged on a ZEN Zeiss spinning disk confocal microscope (Thornwood, NY) and analyzed using the ZEN software. Two regions of interest (ROI) in the perilesional area of one coronal section per brain were analyzed over 8 optical plains, at 0.49µm intervals (Supplementary Figure 7).

*Foot Fault Task*

Mice were placed on a 1 cm x 1 cm elevated grid and allowed to explore for 3 minutes. The total number of steps and slips (faults) per forepaw were counted. We then calculated the % fault slippage using the following equation: $\frac{\left( contralateral faults \right)-\left( ipsilateral faults \right)}{total steps}\times100$ to establish functional deficits or the % fault difference using the following equation: $\left( \left( \frac{Contralateral faults}{Contralateral steps} \right)-\left( \frac{ipsilateral faults}{ipsilateral steps} \right) \right)\times100$ to assess long term deficits and functional recovery. Using % fault difference removes the assumption that mice take equal number of steps between each paw.

*Cylinder Task*

The cylinder task is used to measure forepaw preference in normal mouse rearing behavior. Mice were placed inside an elevated plexiglass cylinder for 4 minutes starting after the first paw contact with the wall of the cylinder. Paw “touches” (contact with the cylinder wall to provide support) were counted per paw, and a % paw preference score was calculated using the following formula: $\frac{Contralateral Paw Touches-Ipsilateral Paw Touches}{Total Paw Touches}\times100$. Simultaneous touches with both paws were counted as “both” and included in the “Total Paw Touches” calculation. Only animals exhibiting a functional deficit greater than 10% ipsilateral paw preference and with > 10 total paw touches were included in our analysis (drNPCs+Vehicle = 9/16; Vehicle alone = 9/21).

*Detailed Statistical analysis*

In establishing the functional deficits of SCID/Beige mice, for the short term (4 days post-stroke) foot fault we used a repeated measures two-way ANOVA, for the long-term deficits in the foot fault we used a repeated measures one-way (time) ANOVA. Unilateral deficits in the foot fault task were analyzed using repeated measures two-way ANOVA. All tests to establish deficits in SCID/beige mice were conducted with a Sidak post-hoc test. drNPC survival and proliferation was analyzed using a two-way ANOVA (day post-stroke, vehicle) to compare between vehicles at each time point and between survival at each time point for each vehicle, followed by a Sidak post-hoc test. Functional recovery in the foot fault and cylinder tasks were conducted using repeated measures, two-way ANOVA (compared to baseline) with a Sidak post-hoc test. We used a repeated measures generalized linear model for the foot fault test (our primary outcome measure) and identified a within-subjects [cell transplant] $\times$ [time] interaction, which we further analyzed using a repeated measured two-way ANOVA with a Sidak post-hoc test. Analysis of extent of gliosis (GFAP) and lesion volumes was conducted with an unpaired T-test when comparing between day 4 and day 32 stroke only (untreated) brains and a two-way ANOVA with a Sidak post-hoc test when comparing between treatment groups (vehicles and transplants). A Fisher’s exact test and chi-square test were used to determine any relationship between transplanted cell survival (brains with drNPCs or no drNPCs at 32 days post-stroke) and transplant vehicle. Comparisons between two groups (drNPCs vs Vehicle) were conducted using a two-tailed unpaired T-test. All correlations were calculated by conducting a bivariate Pearson’s correlation (two-tailed) or a Point-Biseral correlation (two-tailed) between variables of interest. Data were tested for normality using a Shapiro-Wilk Normality Test (confidence interval = 0.95) where deemed necessary.

**Supplementary Figures:**

***Supplementary Figure 1:*** *IHC analysis of Oct4 on human embryonic stem cells*

*a) hESCs express the pluripotency marker Oct4, serving as a positive control for data presented in Figure 1. b) Secondary antibody-only control shows antibody specificity, as no expression is detected. Scale bars = 100 µm.*

***Supplementary Fig 2: ET-1 stroke results in functional deficits and tissue damage in SCID/Beige mice***

**a)** SCID/Beige mice exhibited functional deficits in the foot fault test at day 4 post-stroke compared to naïve (unlesioned) controls. **b)** Mice exhibited deficits at 8 days post-stroke that persisted up to 32 days post-stroke compared to baseline levels in the foot fault test. **c)** Analyzing the performance of each paw (from B) separately shows that the contralateral paw was significantly impaired compared to the performance of the ipsilateral paw on the foot fault test. **d)** Immunohistochemistry for GFAP^+^ expression reveals the presence of a glial scar (or gliosis) and a physical cavity in the ipsilesional cortex but not the contralesional cortex at 4 and 32 days post-stroke. **e)** The lesion volume (which is composed of GFAP^+^ gliosis and physical injury to the tissue, including the cavity) is significantly reduced from 4 days post-stroke to 32 days post-stroke in ET-1 stroke injured mice. Gliosis and injury are also significantly reduced from 4 to 32 days post-stroke. ***(b, c)*** *4 animals were excluded because they did not have a deficit;* ***(e)****: n=5 per timepoint;* *Data are represented as mean ± SEM,(****d****) dashed lines = extent of physical injury boundary, dashed lines = extent of glial scar boundary, CX = cortex, CC = corpus callosum, scale bars = 500 µm, * = p<0.05.*

******

***Supplementary Fig 3: drNPC transplants promote recovery regardless of recipient sex and in the cylinder test***

***(a)*** Male and female transplant recipients display similar impairments post-stroke and functional recovery by day 32. w=male with vehicle different from baseline, x=female with vehicle different from baseline, y=male with drNPCs different from baseline, z=female with drNPCs different from baseline. Data is presented as mean ± SEM (**b)** When exploring functional recovery in the cylinder test, mice that did not exhibit an ipsilateral paw preference greater than 10% after stroke and prior to treatment (n=18) were removed from the study. Mice that received drNPC transplants recovered back to baseline levels by day 32 in the cylinder test, whereas those that received vehicle only injections remained impaired. The two groups were not significantly different from each other at any time point, however. *a=Vehicle different from baseline, b = drNPCs different from baseline, p<0.05*

******

***Supplementary Fig 4: Gliosis volume is strongly correlated with Maximal GFAP^+^ Area***

Once all sections stained with GFAP were measured, the 20µm section exhibiting the maximal GFAP^+^ area was identified in each brain. There is a strong, positive correlation (*r = 0.889, n = 35, p<0.001)* between the total measured gliosis volume and the maximal GFAP^+^ area (in a 20 µm section) per brain, as was determined using using regression analysis.

******

***Supplementary Fig 5: Transplant vehicle has no effect on tissue outcomes****.*

**a)** There was no significant difference in the maximal GFAP^+^ area between vehicles in either transplant or vehicle only treated groups. n>7 for all groups. **b)** There was no significant difference in lesion volume between vehicles in either transplant or vehicle only treated groups. n=4 for all groups
